# Operational efficiency and sustainability of vector control of malaria and dengue: descriptive case studies from the Philippines

**DOI:** 10.1186/1475-2875-11-269

**Published:** 2012-08-08

**Authors:** Henk van den Berg, Raman Velayudhan, Antonietta Ebol, Ben HG Catbagan, Romulo Turingan, Marisol Tuso, Jeffrey Hii

**Affiliations:** 1 Laboratory of Entomology, Wageningen University, P.O. Box 8031, 6700EH Wageningen, The Netherlands; 2 Vector Ecology and Management, Department of Control of Neglected Tropical Diseases, World Health Organization, Geneva, Switzerland; 3 Center for Health Development, Davao City, Philippines; 4 City Health Office, Mati, Philippines; 5 Center for Health Development, Tuguegarao, Philippines; 6 Movement Against Malaria Programme, Pilipinas Shell Foundation, Inc., Butuan, Philippines; 7 World Health Organization Representative's Office in the Philippines, Manila, Philippines

**Keywords:** Community participation, Elimination, Empowerment, Health systems, Integrated vector management, Inter-sectoral collaboration, Micro-stratification

## Abstract

**Background:**

Analysis is lacking on the management of vector control systems in disease-endemic countries with respect to the efficiency and sustainability of operations.

**Methods:**

Three locations were selected, at the scale of province, municipality and barangay (i.e. village). Data on disease incidence, programme activities, and programme management were collected on-site through meetings and focus group discussions.

**Results:**

Adaptation of disease control strategies to the epidemiological situation per barangay, through micro-stratification, brings gains in efficiency, but should be accompanied by further capacity building on local situational analysis for better selection and targeting of vector control interventions within the barangay. An integrated approach to vector control, aiming to improve the rational use of resources, was evident with a multi-disease strategy for detection and response, and by the use of combinations of vector control methods. Collaboration within the health sector was apparent from the involvement of barangay health workers, re-orientation of job descriptions and the creation of a disease surveillance unit. The engagement of barangay leaders and use of existing community structures helped mobilize local resources and voluntary services for vector control. In one location, local authorities and the community were involved in the planning, implementation and evaluation of malaria control, which triggered local programme ownership.

**Conclusions:**

Strategies that contributed to an improved efficiency and sustainability of vector control operations were: micro-stratification, integration of vector control within the health sector, a multi-disease approach, involvement of local authorities, and empowerment of communities. Capacity building on situational analysis and vector surveillance should be addressed through national policy and guidelines.

## Background

Vector-borne diseases cause a major burden in the Philippines. Main diseases are malaria, dengue, lymphatic filariasis, schistosomiasis and Japanese encephalitis [1,2]. The Philippine Department of Health and the National Malaria Control Programme aim to eliminate malaria from the Philippines by 2020 [3]. Also, the country is on its way to elimination of lymphatic filariasis [4]. The dengue control programme has been decentralized, with guidelines provided by the national level but operations depend mostly on funds and decision-making at the municipal level [5].

Vector control has the potential to play an important role in reducing transmission of these diseases and in reaching critical low levels of vectorial capacity required for elimination of disease [6-9]. To be effective, the methods of vector control should be adapted to the ecology, behaviour, and insecticide susceptibility of vector populations, and personal protection methods should be tailored to people's habits and preferences. To be efficient, vector control should be targeted where and when people are most at risk. Well-adapted and well-targeted vector control strategies will ensure the efficient use of resources and contribute to the effective reduction of the disease burden. Moreover, malaria, dengue, lymphatic filariasis and Japanese encephalitis are all transmitted by mosquitoes and, in areas where more than one disease is co-endemic, these diseases are potentially controlled by the same interventions or strategies.

The technical and operational sustainability of vector control is of major concern, given the threat of insecticide resistance [10-12], and given the current dependence on external funding - particularly in malaria control. This situation could be improved by the integrated use of alternative methods of vector control, an effective insecticide resistance management strategy, and the integration of vector management strategies into existing systems and structures.

Numerous reports are available on the effectiveness and costs of individual vector control interventions, with some of these interventions having an application value in diverse epidemiological settings [13,14]. However, analysis and documentation has been largely lacking on how vector control is being managed. Specifically, there is an urgent need to study how vector control is being planned and implemented with respect to the efficiency and sustainability of operations.

The World Health Organization promotes the principles and approaches set out in the strategic framework on integrated vector management (IVM) to improve the efficacy, cost-effectiveness, ecological soundness and sustainability of vector control [15]. A recent survey among countries endemic or at risk of vector-borne diseases showed that 62% of 110 countries reported having a national policy on IVM in place [16]; hence, the political endorsement to improve vector control systems already exists. Nevertheless, further advocacy is needed to inform decision-makers whether a re-orientation or re-organization of their vector control systems will pay off in terms of health, social and economic benefits [17]. In this regard, case studies are a powerful advocacy tool because they can demonstrate benefits through real-world examples.

This study was initiated on the assumption that lessons could be learnt about operational efficiency and sustainability by studying vector control systems established at different levels of public administration because at each level, distinct conditions for decision-making and integration would apply. The outcomes were evaluated in relation to the five key elements laid-out in the framework on IVM: evidence-based decision-making; an integrated approach; collaboration within the health sector and with other sectors; advocacy, social mobilization and legislation; and capacity building [15]. As the study of vector control systems is complicated by contextual and partly unknown circumstances, the analysis was centred on the general lessons learnt in order to deduce their significance for formulating policy and guidelines.

## Methods

Three locations were selected, at different administrative levels, i.e. province, municipality and barangay; a barangay is the smallest administrative division in the Philippines and is equivalent to a village.

The first case study location, Cagayan Valley in north-eastern Luzon, comprises the provinces of Isabela and Cagayan (combined population 2.4 million). This area was selected as an example of a malaria elimination programme showing promising results in the reduction of malaria cases. The programme in Cagayan Valley was implemented under the National Malaria Control Programme, with main support from the Global Fund. The second case study location was the municipality of Mati City (population 128,000), in Davao Oriental province in south-eastern Mindanao. This location was selected because of its known local efforts in developing an epidemic response system for vector-borne disease control. The third case study location, the barangay of Simbalan (population 3,800), in Buenavista municipality, Agusan del Norte province in northern Mindanao, was selected as a known example of a barangay with active involvement of local leaders and communities in vector control.

The case studies were conducted on site in June 2011. Data on programme achievements in terms of training, supervision and interventions and data on disease incidence rates were gathered through meetings at health offices at regional, provincial and municipal level. Focus group discussions were held in selected municipalities and barangays with local government officers, barangay health workers and members of civil society to obtain qualitative data on the recent history, structure, organization and management of local programmes.

## Results

### Case 1: Cagayan Valley

The main malaria vectors in Cagayan Valley, as in most of the Philippines, are *Anopheles flavirostris *and *Anopheles maculatus*, which breed at the edges of streams in foothills, especially near human habitation [18-20]. In 2005, training and infrastructure development for barangay microscopy centres and rapid diagnostic test (RDT) sites in remote areas have improved the quality of malaria detection and diagnostic services, often through integration with existing community mechanisms, e.g. by involving midwives. Also, a system of regular reporting and data management of malaria cases has been in place.

These malaria incidence data have enabled micro-stratification of malaria epidemiology, with the barangay as stratification unit. The micro-stratification, conducted in 2010, categorized individual barangays as having 'stable transmission', 'unstable transmission', 'sporadic transmission' or being 'malaria prone', defined on the basis of monthly patterns of malaria transmission using criteria presented in Table 1. The majority of cases were infected with *Plasmodium falciparum*. The purpose of the micro-stratification, to be updated every three years, was to aid programme managers in their planning, choice of interventions and efficient use of resources for case detection and vector control in accordance with the disease situation in each barangay.

**Table 1:** Micro-stratification of malaria epidemiology

Item	Category			
	
	'Stable transmission'	'Unstable transmission'	'Sporadic transmission'	'Malaria prone'
Categorization criteria	String of 6 or more months with continu-ous transmission in the period 2007-09	String of 2-5 months with continuous transmission in the period 2007-09	At least one indigenous case in the period 2005-09	No indigenous case in the period 2005-09
Number of barangays:				
Cagayan Province	15	77	164	565
Isabela Province	6	26	58	745
Objective	Malaria control	Pre-elimination	Elimination	Maintenance
Strategy:				
Clinical surveillance	Passive Case Detection (PCD)	Active Case Detection (monthly); PCD	Mass blood survey (annually); case investigation; PCD	Case investigation; PCD
Diagnosis and treatment	Microscopy, Rapid Diagnostic Testing (RDT); treatment of confirmed cases	Microscopy, RDT; treatment of confirmed cases	Microscopy; treatment of confirmed cases	Microscopy; treatment of confirmed cases
Long-lasting insecticidal nets (LLIN)	100% coverage of households	100% coverage of households	100% coverage of households	Only in case of epidemic
Indoor residual spraying	When LLIN gives no improvement after 1 year	Only in case of epidemic or displaced populations	Only in case of epidemic or displaced populations	Only in case of epidemic
Environmental management	Where appropriate	Where appropriate	Where appropriate	Where appropriate
Social mobilization	Health promotion	Health promotion	Health promotion	Health promotion

The unit of stratification was the barangay. Where the radius of a barangay was more than 5 km, the sub-barangay, or *sitio*, was used as stratification unit

The results showed that the distribution of malaria was highly focal, with 'stable transmission' occurring in less than 1% of the barangays and the majority of barangays being classified as 'malaria prone' (Table 1). To each stratification category, a unique strategy of malaria control or elimination was applied. For example, barangays with 'stable transmission' were given greater emphasis on vector control, whereas barangays with sporadic malaria were given emphasis on disease surveillance to detect any remaining cases or re-introductions.

Over the period 2005-2010, the implementation of the tailor-made malaria control strategy was accompanied by a remarkable decline in malaria cases in both provinces (Table 2). The trend is a strong indication that the strategy with its package of interventions has been effective. The decline was more drastic in Isabela province than in Cagayan province. The available information does not allow for teasing out the contribution of the individual interventions. With current knowledge, it remains largely unclear whether the combination of indoor residual spraying (IRS) and long-lasting insecticidal nets (LLIN) provides additional benefits compared to LLINs alone [21]. If IRS should continue as additional intervention to LLINs, then it should be with an insecticide with a different mode of action [22].

**Table 2:** Malaria cases in relation to vector control interventions

	Isabela Province				Cagayan Province			
		Intervention				Intervention		
				
Year	Cases	ITN	LLIN	IRS	Cases	ITN	LLIN	IRS
2005	1,444	23,177	31,857	1,215	1,472	21,221	12,806	6,081
2006	833	23,626	14,974	4,784	1,139	34,566	11,954	5,726
2007	869	51,774	22,230	4,673	1,162	57,227	20,800	11,425
2008	239	65,244	0	7,034	772	47,433	2,680	13,541
2009	132	21,597	35,078	6,210	541	30,175	38,326	16,499
2010	74	2,011	32,293	9,756	435	1,808	181,516	22,745

ITN, number of conventional insecticide-treated nets distributed or re-treated; LLIN, number of long-lasting insecticidal nets distributed; IRS, number of houses with residual spraying

The micro-stratification helped not only to improve the efficient use of resources; it also stimulated the participation and support from barangay leaders. It was evident during the visits to the barangays that chiefs and councillors were cognizant about the programme and its local achievements, through their liaison with the barangay microscopy centre, and that they had been personally involved in the organization of bed net distribution campaigns.

Despite the demonstrated benefits, there is potential to improve the strategy. In particular, the current model only considers where cases of malaria are diagnosed but does not determine where transmission occurs; who is most at risk; or which other vector-borne diseases are locally prevalent. If these ecological and sociological elements are incorporated into the micro-stratification, this could increase the programme's effectiveness and sustainability in a decentralized health system.

This is illustrated by comparing the situation in two barangays visited: Antagan (Tumauini, Isabela province), with a population of 2,675, and Mabuno (Gattaran, Cagayan province), with a population of 2,640. Both barangays had been malaria hotspots (Table 3). Microscopy centres were newly established in barangay centres in 2005 and RDT sites were added in outlying parts of the barangay in 2006 and 2007. Consequently, it is probable that the actual number of cases has been under-estimated in 2005. Malaria cases in Antagan had dropped after 2006, but cases did not decline in Mabuno barangay, despite full coverage with vector control interventions.

**Table 3:** Malaria cases in relation to vector control interventions

	Antagan barangay				Mabuno barangay			
		Intervention				Intervention		
				
Year	Cases	ITN	LLIN	IRS	Cases	ITN	LLIN	IRS
2005*	5	75	0	0	29	0	579	0
2006	43	130	157	120	28	1,105	70	27
2007	10	165	265	0	74	829	0	250
2008	4	181	0	0	49	1,323	98	400
2009	2	0	786	165	12	195	1,108	1,203
2010	1	0	881	300	50	0	0	536

ITN, number of conventional insecticide-treated nets distributed or re-treated; LLIN, number of long-lasting insecticidal nets distributed; IRS, number of houses with residual spraying

* Malaria cases were probably under-estimated in 2005

Close examination of case reports revealed that in Antagan, the malaria cases were mostly male (92%), from 11 to 30 years (77%), occupational loggers (53%) or farmers (32%), and that 65% of all cases occurred during the months of June and July, coinciding with the planting season of rain-fed crops in the hills. Men staying out in the hills to plant and cultivate these crops during the rainy season, or to log trees, are apparently at risk to be infected with malaria, because these are the known habitats where the main mosquito vectors breed. Hence, it is likely that the remaining malaria transmission in Antagan occurred not within the barangay proper but out in the hills. The causes of the recent drop in cases (e.g. due to the effect of health promotion, or due to a provincial ban on logging enacted in 2010) need further investigation.

Conversely, in Mabuno, malaria cases did not decline even with high coverage of LLINs and IRS. Here, the majority of cases were indigenous people, living in make-shift houses in the foothills, which were areas not covered under recent LLIN-distribution campaigns, and reporting to the barangay health centre for treatment when they were ill. This suggests that transmission has continued among indigenous people living unprotected from infectious bites outside of the barangay proper.

The examples of Antagan and Mabuno indicate that information other than basic epidemiological data was important for evidence-based decision-making. Entomological data and information on personal protection behaviour among those staying in the foothills could help adapt the malaria control strategy to achieve further reductions in disease incidence. For example, increased attention could be paid to mobile indigenous populations and temporary agricultural workers, developing appropriate control methods (e.g. insecticidal hammocks), and targeting the interventions at times and places when and where these groups are most at risk of malaria transmission. Also, entomological surveillance to confirm the absence of malaria vectors within the barangay proper will help improve the efficient allocation and use of resources for vector control and personal protection.

Summing up, the case from Cagayan Valley demonstrates a model for planning and adapting malaria control strategies to the epidemiological situation in each barangay. The benefits of micro-stratification were three-fold: it resulted in more efficient use of resources; it allowed for more in-depth understanding about malaria transmission within the barangay; and, it stimulated the involvement of local leaders. An important lesson is that this model could be improved further by collecting entomological data and determining which sections of society are most at risk of malaria transmission.

### Case 2: Mati City

**Figure 1:**
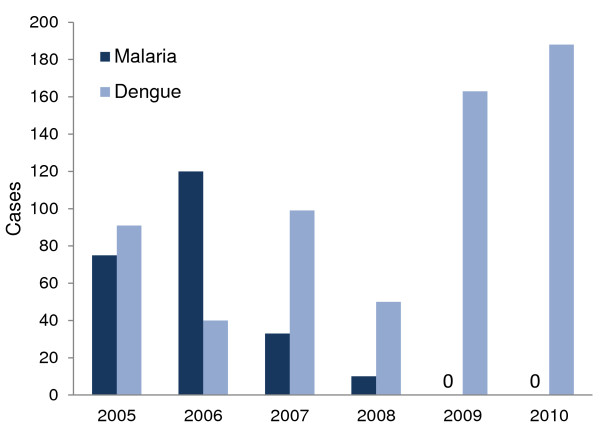
**Indigenous cases of malaria and dengue in the municipality of Mati City**.

The municipality of Mati City has been affected by both malaria and dengue. Malaria control has been well structured through the National Programme, with support from the Roll Back Malaria Project and Global Fund for case finding, treatment, and vector control through LLINs and IRS as well as capability building for local health staff and volunteers. These efforts were accompanied by a decline in malaria prevalence during the period 2005-2010 (Figure 1), noting that case detection was not yet fully operational in 2005. No more cases of indigenous transmission were reported after 2008, with the only reported cases being imported from other municipalities or by fishermen visiting from abroad (Municipal Health Office, unpublished data, 2011).

In contrast, the prevalence of dengue, transmitted by *Aedes *spp. mosquitoes, has been on the increase (Figure 1). Dengue control has been less structured and has received far less financial support than malaria control. In the absence of effective medication against dengue, prevention through vector control is the only option for control of this disease.

In 2006, a training programme was conducted for 23 barangay health workers on communication and social mobilization for dengue prevention and control, based on methods described by Parks and Lloyd [23]. Health promotion was conducted on four desired behaviours regarding treatment seeking and vector breeding that had been identified for advocacy: (i) if fever cases are not relieved after two days of medication, consult a health facility; (ii) cover all water containers inside and outside at all times; (iii) clean all water containers before refilling at least once a week; and (iv) cover or drill holes in used tyres to prevent water accumulation. The health workers subsequently conducted monthly activities of health promotion and dengue vector surveillance in four barangays selected as dengue hotspots. The number of water-holding bodies and those with presence of larvae of *Aedes *spp. were recorded in 100 sampled households per barangay. Samples of mosquito larvae were taken to the laboratory for identification.

The continued burden of dengue, however, led City health authorities to revisit and intensify the communication strategy in 2010. A malaria-dengue task force was established, with a mandate to detect, and respond to, the incidence of malaria, dengue and any emerging vector-borne disease. Maps of individual barangays were used to locate cases, stratify incidence and plan response actions. Also, a rapid deployment team was recruited to strengthen case detection and outbreak response in the barangays, by following up suspected dengue cases through visits, carrying out case surveillance at health centres, and supporting health promotion in hotspot barangays. The task force and its rapid deployment team had been funded by a locally operating mining company as a form of public-private collaboration. These funds, initially ear-marked for malaria control, were available as in-kind support for dengue control.

This support for dengue was, however, coming to an end in 2011 and, in response, the City health authorities had taken two measures to integrate vector-borne disease control within the health infrastructure. First, in barangays that were dengue hotspots, the existing 'barangay health emergency response' teams were trained to adopt dengue prevention as part of their job description. These teams, present in every barangay since they were enacted nationwide in 2003 to stop the spread of severe acute respiratory syndrome (SARS), began to organize weekly clean-up drives and conduct vector larval surveillance at monthly intervals. Second, a disease surveillance unit was created to enact and coordinate weekly reporting of cases of all notifiable diseases, including vector-borne diseases, from the city's two hospitals and from all 26 barangay health centres. These two measures helped safeguard the regular allocation in City budgeting for the detection and control of vector-borne diseases, notably dengue. In addition, in 2011, the City mayor's office began sponsoring the rapid deployment team, driven in part by the economic burden of dengue, due to costs incurred by the mayor's office for transport, treatment and referral of dengue cases to specialized hospitals.

In the event of an increase in dengue cases or a local dengue outbreak, the relevant agencies were alerted to respond. The response consisted of intensified health promotion and weekly rounds of vector surveillance and concomitant source reduction in 100 houses in the vicinity of detected cases. The weekly rounds were repeated for up to 10 times, until the 'house index' (i.e. % of houses being positive for dengue breeding) dropped below the locally set threshold margin of 2-5% and the 'Breteau index' (i.e. the number of positive containers per 100 houses) dropped below the value of 20 [24]. Hence, entomological monitoring provided a direct feedback for decision-making on interventions. Also, active case surveillance and campaigns to treat curtains with pyrethroids against dengue vectors were implemented. Larviciding was only occasionally carried out but space spraying was not conducted. Advocacy and communication was provided by the City health office through regularly produced updates on the dengue situation for dissemination in hotspot barangays. A remaining challenge is to increase the participation and programme ownership of the community at large in vector control [25,26].

There were other partners in vector control. The department of education created their own vector control task force with monitoring by brigades at schools. Moreover, barangay chiefs and councillors, having been among those trained on dengue prevention, provided support for health promotion and clean-up drives, issued permits to search people's compounds for vector breeding sites and, in one instance, formulated policy. After four child deaths due to dengue in one sub-barangay in 2009, the local councillor introduced a resolution that households must pay a fine of 50 pesos if *Aedes *larvae are found on their compound. The resolution was adopted by the entire barangay, and the generated income went into a trust fund to treat dengue patients.

Hence, the case of Mati shows how in-kind contributions for malaria control benefited dengue control activities through a joint malaria-dengue system of detection and vector control response. Even though dengue was the only prevalent vector-borne disease at the time of the case study, the system was prepared to respond to outbreaks of malaria and other diseases, thus increasing the efficiency as compared to single-disease systems. To safeguard the operational sustainability, the detection and response system was integrated within the health infrastructure. Cases of dengue fever dropped from 185 in 2010 to 108 in 2011; the coming years will show whether the strategy leads to a further reduction in disease cases and whether the political commitment can be sustained.

### Case 3: Simbalan barangay

Buenavista municipality has been relatively free from malaria, with the exception of a pocket of 'stable transmission' of malaria around Simbalan, a barangay composed of a number of scattered sub-barangays or *sitios *in a poorly accessible mountainous area. Sporadic cases of lymphatic filariasis were reported in the past, but dengue has been absent.

Through the externally funded National Malaria Control Programme, Simbalan established a functional microscopy centre in 2005, with seven RDT sites as satellites in the outer *sitios*, and in that year the number of confirmed malaria cases (predominantly *P. falciparum*) was determined at 490 (i.e. incidence rate of 129 per 1,000 population). In recent years, full coverage of the population with insecticidal nets has been achieved, whereas IRS was started in 2010 in the western-most part of the barangay where transmission risk through imported cases was considered to be highest. The number of malaria cases dropped to zero (Table 4). Mass blood surveys have been conducted to detect any remaining cases. The drastic decline in malaria cases indicated that the control effort paid off.

**Table 4:** Malaria cases in relation to vector control interventions

	Simbalan barangay				Buenavista municipality			
		Intervention				Intervention		
				
Year	Cases	ITN	LLIN	IRS	Cases	ITN	LLIN	IRS
2005	490	442	0	0	579	749	0	0
2006	23	0	0	0	38	25	0	0
2007	0	0	0	0	43	400	0	0
2008	8	0	0	0	8	0	0	0
2009	2	0	2,442	0	2	0	n/a	0
2010	0	0	1,477	113	0	0	n/a	113

ITN, number of conventional insecticide-treated nets distributed or re-treated; LLIN, number of long-lasting insecticidal nets distributed; IRS, number of houses with residual spraying; n/a, data not available

Malaria control in Simbalan benefited from strong local leadership by the barangay chief and councillors. From 2005, five types of local initiatives on malaria control emerged. First, a barangay action committee on malaria, with participation from local officials, health workers, teachers, and several community-based groups, was established to plan and coordinate malaria control. Initially, seed-funding assistance and technical guidance had been provided to establish this committee. Second, under the auspices of this committee, an anti-malaria brigade of volunteers was formed in every *sitio *with the aim of implementing vector control at monthly intervals by clearance of streams, improvement of the water flow and removal of overhanging vegetation. These brigades also assisted in health promotion, LLIN surveys, mass blood surveys, rearing and releasing of larvivorous fish for vector control and, in some instances, intra-domiciliary spraying.

Third, through a small-scale public-private partnership, the local motorbike-taxi association provided transportation services, usually for free, in support of malaria control. These services, which included the transport of patients, blood slides and reports, have been vital in view of the isolated location of the barangay. Fourth, regular house-to-house visits were carried out by so-called 'personal sellers', to promote and monitor the utilization and maintenance of LLINs among residents. These volunteers were trained on health promotion by the provincial health office, and assisted in case finding, blood-smear collection, and mass blood surveys. Fifth, health education on malaria transmission and vector control was conducted in schools. Each of these local initiatives benefited several of the components of malaria control and, thus, enhanced or complemented the interventions by the health sector (Table 5).

**Table 5:** Local initiatives benefiting malaria control

Local initiatives	Malaria control component			
	
	Detection, diagnosis	Case treatment	Vector control	Health promotion
1. Barangay action committee	+	+	+	+

2. Anti-malaria brigades	+		+	+

3. Transportation services	+	+		

4. House-to-house visits			+	+

5. School education programme			+	+

Indicated are the malaria control components incorporated in each local initiative

A pivotal role in co-ordinating malaria control activities in Simbalan has been played by the trained microscopist and barangay health worker. She supervised the RDT sites, mobilized mass blood surveys where recent cases have been found, and organized surveys on the needs, quality and utilization of bed nets. On her initiative, recipients of LLINs had to sign a declaration stating their accountability to use and maintain the nets for their intended purpose. Her reports on the results of surveys and other activities were send to the municipal health office and were also shared within the barangay, as a means of feedback to those involved in malaria control activities.

Clearly, the barangay, its community, schools and taxi association have taken ownership of malaria control through their engagement in the planning, implementation and evaluation of interventions, though still requiring external funding support. The barangay action committee pro-actively developed its own vision statement to become independent of external resources for malaria control, indicating a commitment to sustain malaria control and elimination. Their data on case detection and bed net use have served as a basis for action planning. From the onset, the effort in Simbalan focused on malaria control, but prevention of other vector-borne diseases, such as lymphatic filariasis, could potentially be incorporated within the same strategy.

## Discussion

The case studies provided practical examples of the management of disease vector control at the level of province, municipality and barangay. Some aspects in the case studies, e.g. micro-stratification, were part of a national strategy, and were therefore representative for the country. Other aspects, e.g. health sector integration, were products of local contextual variables and decentralized health systems, and are not necessarily representative of the country because the case study locations had not been randomly selected. The results are discussed in accordance with the key elements of the IVM framework (Table 6).

**Table 6:** Key elements of IVM represented in the three cases

	Key element	Cagayan Valley (province level)	Mati (municipal level)	Simbalan (barangay level)
1	Evidence-based decision making	Micro-stratification as the basis for tailor-made strategies per barangay *But: *entomological and human behavioural data not collected	Case detection, mapping and vector surveillance as a basis for response action *But*: methods of vector surveillance should be improved	Case detection and evaluation of bed net utilization as basis for local action planning *But*: evidence on environmental management lacking

2	Integrated approach		Multi-disease strategy of detection and response; combination of vector control methods	Combination of chemical and non-chemical vector control methods

3	Collaboration within the health sector and with other sectors	Local government involvement	Re-orientation of barangay health emergency response teams; integrated disease surveillance unit; some collaboration with education and mining sectors; local government involvement	Public-private partnership; local government involvement

4	Advocacy, social mobilization and legislation	Health promotion	Campaigns on behavioural change; clean-up drives; local legislation on vector control	Local initiatives on malaria control; local programme ownership

5	Capacity building	Training on detection and diagnosis; infrastructure *But*: training on vector surveillance and situational analysis lacking	Training on detection and diagnosis; infrastructure; training on behavioural change and vector surveillance	Training on detection and diagnosis; infrastructure *But*: training on vector surveillance lacking

Evidence-based decision-making was apparent in each case example, contrasting shortcomings noted in an earlier study [5]. In Cagayan Valley, adaptation of disease control strategies to the epidemiological situation per barangay, through micro-stratification, brought obvious gains in efficiency of operations at provincial level. The model of micro-stratification, which has been applied in all provinces covered by the National Programme on malaria elimination, could potentially be enhanced by incorporating data on vector-borne diseases other than malaria to determine where diseases coexist as a basis for coordinating vector control action. Moreover, the example of Cagayan Valley revealed that micro-stratification should be accompanied by further capacity building on local situational analysis to elucidate where plasmodia transmission takes place (e.g. in the foothills) and who is most at risk (e.g. mobile indigenous populations and temporary workers). This would lead to better selection and targeting of vector control interventions within the barangay.

In this respect, the examples at lower levels of administration, i.e. in Mati and Simbalan, have been reassuring. Here, decisions on vector control were locally adapted in accordance with data on case detection, vector surveillance (Mati) and evaluation of bed net utilization (Simbalan). Hence, surveillance data were readily utilized. Nevertheless, gaps remained in the evidence base needed for decision-making on vector control. Particularly, data on the cost-effectiveness of stream clearing against *Anopheles flavirostris *[20], and on combinations of interventions, such as IRS in areas of high LLIN coverage [21,27], were lacking. Moreover, there is prospect for improving the monitoring and targeting of dengue vector populations in the example of Mati, e.g. by monitoring the number of *Aedes *pupae instead of the presence of larvae for identification of the most productive breeding sites [28], and by targeting based on the number of pupae per person as a measure of transmission risk [24].

An integrated approach to vector control, aiming to improve the rational use of resources, was evident in the case of Mati where elements of a multi-disease strategy of detection and response had been established (Table 6). Also, the integrated use of vector control methods was apparent in the case examples, even though the combination of selected methods needs to be supported by further evidence.

Collaboration on vector control can improve operational efficiency within the health sector and lead to reduced disease risks in other sectors. Collaboration within the health sector was apparent from the involvement of barangay health workers in vector-borne disease control in each case study, but was most obvious in Mati, where the job descriptions of barangay health emergency response teams were modified and staff reoriented towards vector control and where a central unit was created for weekly surveillance of notifiable disease cases, including vector-borne disease cases (Table 6). Health agencies and barangay health workers collaborated closely with local government units and, in the case of Mati, also with the education department and mining sector, on disease vector control. Nonetheless, there is prospect for involving other relevant sectors (e.g. education, environment and agriculture) in vector control.

Advocacy and social mobilization were common features in each case study (Table 6). Specifically, in Mati, recurring campaigns were carried out in barangays that were dengue hotspots to achieve behavioural change on vector control. The degree to which communities participated in vector control in the three cases ranged from passive, active, to empowerment [29]: communities were taught to comply with programme interventions on the use of LLINs (Cagayan Valley), they actively conducted source reduction for dengue prevention (Mati), and took control over a local programme (Simbalan).

Capacity building is a major challenge to the improvement of vector control systems. The case examples indicate that a national strategy for capacity building on detection and diagnosis of malaria was in place, but competencies on situational analysis and vector surveillance appeared to be inadequate. These aspects should be addressed in national policy and guidelines.

Considering the current reliance on external funding support, the operational sustainability of vector control remains uncertain. It will be particularly challenging to sustain vector control during and beyond the elimination of disease, after the public health problem has diminished [6-8]; however, access to additional emergency funds in the event of outbreaks has been noted [5]. With recent support for malaria elimination, malaria cases have declined in the Philippines and several other Southeast Asian countries [2]. Conversely, dengue has increased in importance in Southeast Asia but has so far failed to attract major international funding [30-32]. This dual pattern suggests the need for a better coordinated, multi-disease strategy of vector control wherever these diseases are co-endemic. In the case of Mati, health authorities made opportunistic use of complementary effects between their malaria and dengue control programmes. Hence, countries and funding agencies should support capacity-building on vector surveillance and control that is not disease-specific but includes vectors of other prevalent, co-endemic or emerging diseases.

The case studies have suggested several other mechanisms to sustain vector control: integration within the health sector, involvement of local authorities, and empowerment of communities. In Mati, financial support for dengue control was coming to an end, which prompted the municipal health authorities to incorporate dengue control within their health system, utilizing local infrastructure and safeguarding regular allocation of funds for vector-borne disease control in local budgeting. Yet, many municipalities with dengue prevalence may lack such resources and, thus, sufficient national funds should be allotted to the control of this neglected disease.

The engagement of barangay leaders and existing community structures helped mobilize local resources and voluntary services for vector control. Barangay authorities and the community in Simbalan were actively involved in the planning, implementation and evaluation of malaria control actions, indicative of local programme ownership. Moreover, their vision statement to become independent from external resources for malaria control is particularly relevant in the context of malaria elimination because continued commitment to surveillance and outbreak response will be needed [33]. Nevertheless, the challenge remains for its leaders to maintain the commitment to malaria prevention when in future the memory of the past malaria burden may fade away. Several supporting factors were apparent in Simbalan: provision of training and guidance by health agencies; local government leadership; and a spirit of community volunteerism, or *bayanihan*, a common tradition in Philippine towns [34]. This suggests that the model of local programme ownership could be replicated in other barangays with strong leadership, provided that adequate training and guidance are given.

## Conclusions

Micro-stratification based on epidemiological data resulted in improved efficiency of disease control operations, but should be accompanied by further capacity building on the selection and targeting of vector control interventions at barangay-level. The case studies suggested several mechanisms to improve operational sustainability of vector control: integration within the health sector, a multi-disease malaria-dengue approach, involvement of local authorities, and empowerment of communities. Local programme ownership on vector-borne disease control could potentially be replicated if adequate training and guidance are given.

## Abbreviations

LLIN: Long-lasting insecticidal nets; IRS: Indoor residual spraying; ITN: (conventional) Insecticide-treated nets; IVM: Integrated vector management; RDT: Rapid diagnostic test; SARS: Severe acute respiratory syndrome; WHO: World Health Organization.

## Competing interests

The authors declare that they have no competing interests.

## Authors' contributions

HvdB, RV, AE, BHGC, RT, MT and JH all participated in field visits and data collection. JH conceptualized the study. HvdB analysed and interpreted the data and drafted and revised the manuscript. JH, RV, BHGC, AE, RT and MT contributed to revising the manuscript. All authors have read and approved the final manuscript.
